# Secure command transmission techniques for industrial remote control

**DOI:** 10.1038/s41598-025-01290-x

**Published:** 2025-05-14

**Authors:** Anas Abu Al-Hija’a, M. Andó, B.J. Szekeres

**Affiliations:** Institute of Computer Science, Faculty of Informatics of Eötvös Loránd University, ELTE, Budapest, Hungary

**Keywords:** Industry 4.0, Remote control systems, Secure code transmission, Industrial Internet of Things (IIoT), Engineering, Mathematics and computing

## Abstract

Operating a factory near a war zone or in a country with an unstable political environment poses significant risks. These risks can be mitigated by managing production lines remotely. To preserve technical knowledge and specific algorithms, it is essential to implement secure, precise, and efficient remote control in industrial applications, especially considering the risks associated with storing operational code on controllers. To address these challenges, we propose a novel technique where executable commands are dynamically transmitted from a Python script to an ESP32-WROOM-32 microcontroller. Unlike conventional methods that preload code onto the controller, this approach interprets, executes, and erases commands immediately after execution, thereby enhancing security and precision. The proposed system was evaluated through a comparative analysis of eleven distinct methods, which varied in command transmission strategies and employed dual-core processing for performance optimization. VPN technology was integrated to enable remote control from geographically distant locations, demonstrating the system’s adaptability for global industrial operations. The results indicate that this method significantly outperforms the traditional on-site approach, where operational code is preloaded onto the microcontroller. Specifically, bundled command transmission combined with dual-core processing proved particularly effective, reducing latency and improving reliability. These findings highlight the robustness of the proposed approach as a secure and flexible solution for modern smart factories.

## Introduction

The advancement of smart factories necessitates the development of secure and efficient industrial remote control systems. These systems enable real-time monitoring and control of manufacturing processes, enhancing productivity and flexibility. However, their increased connectivity introduces vulnerabilities to cyber threats, underscoring the importance of robust security measures. Huo et al. demonstrated how 5G technology enhances low-latency and high-reliability communication, addressing challenges in harsh and dynamic industrial environments, such as coal mines, and ensuring secure and efficient operations^1^. Recent research emphasizes the integration of lightweight authentication mechanisms^2^ and the deployment of Industrial Internet of Things (IIoT) frameworks^3^ to bolster security while maintaining operational efficiency. Implementing these strategies is crucial for safeguarding critical infrastructure and ensuring the resilience of smart manufacturing environments.

Achieving secure, precise, and reliable control in dynamic industrial processes presents significant challenges due to modern industrial systems’ increasing complexity and interconnectivity. Integrating cyber-physical components introduces vulnerabilities that can compromise system integrity and performance. Wang et al. emphasized the importance of structured methodologies to ensure safety, reliability, and precision, particularly in complex environments like mining and manufacturing^4^. Ensuring real-time responsiveness while maintaining stringent security measures requires advanced control strategies capable of adapting to dynamic conditions and mitigating potential threats^5^. Despite these advancements, many current remote-based industrial models rely on preloading operational code onto microcontrollers or other embedded devices^6,7^. While this setup can be sufficient for static or semi-static operations, it limits real-time adaptability and introduces additional security risks, as stored code can be reverse-engineered or exploited by unauthorized actors^5^. Furthermore, the need for frequent firmware updates can be cumbersome in distributed manufacturing sites, particularly those requiring rapid reconfiguration of production lines or enhanced cyber-protection^8^. These shortcomings highlight a critical gap in remote-control solutions: how to design a system that avoids permanently storing proprietary algorithms on local devices, thereby reducing the exposure of sensitive code, while still providing reliable real-time performance over geographically dispersed locations.

Furthermore, while existing remote-control methods offer certain advantages, they often rely on storing operational code locally, posing security vulnerabilities and limiting flexibility^6–8^.We address these limitations in the Discussion section by comparing state-of-the-art solutions and highlighting the need for a dynamic transmission approach.

Our work addresses this gap by proposing a dynamic command-transmission approach that eliminates the need for on-site code storage. Instead, executable commands are pushed from a secure Python-based control script to the ESP32 microcontroller in real time, executed immediately, and erased upon completion. This design mitigates the risk of intellectual property theft or misuse of proprietary algorithms while enhancing production flexibility, factories cannot run without receiving the required commands from the remote controller. Moreover, leveraging VPN technology enables secure transmissions across diverse network infrastructures, ensuring robust cybersecurity. By systematically comparing multiple command-transmission paradigms (i.e., single vs. bundled, single-core vs. dual-core, local vs. VPN), our study demonstrates how ephemeral code execution can outperform conventional on-site approaches under various industrial use cases. Thus, our research not only fills a gap in current industry practices but also provides a template for security-centric, adaptive, and globally scalable remote-control frameworks.

Moreover, high precision in control actions demands robust algorithms to handle uncertainties and disturbances inherent in industrial environments^9^. Addressing these challenges necessitates a multidisciplinary approach, combining control theory, cybersecurity, and industrial engineering insights to develop resilient and efficient control systems^10^.

Traditional on-site control methods, which rely on preloaded operational code on microcontrollers, face several limitations. These include inflexibility in adapting to real-time changes, makes difficulties in updating firmware, and increased vulnerability to security threats due to static code deployment^7^. Such constraints hinder the implementation of advanced control algorithms and the ability to respond dynamically to operational demands^6^, limiting their application in modern, complex systems requiring real-time adaptability^11^.

Recent advancements in microcontroller technology have led to the development of devices like the ESP32-WROOM-32, which integrates dual-core processing capabilities and supports multiple communication protocols. This integration enhances performance and versatility in various applications, including IoT and embedded systems. For instance, the ESP32’s support for protocols such as MQTT, CoAP, HTTP, and XMPP facilitates efficient data transmission in IoT environments^12^. Additionally, its dual-core architecture allows for concurrent processing tasks, improving system responsiveness and efficiency^13^. These features make the ESP32-WROOM-32 a cost-effective solution for developing robust and scalable IoT applications.

Parallel programming is essential in controlling industrial processes, enabling efficient management of complex, real-time operations. By distributing computational tasks across multiple processors, parallel programming enhances system responsiveness and reliability, which is crucial for applications such as real-time monitoring and adaptive control in manufacturing environments^14,15^. This approach allows for the handling of large-scale data and the execution of intricate algorithms, leading to improved performance and productivity in industrial settings^16^.

Efficient control of industrial processes necessitates adaptable methods for handling diverse command transmission types. For instance, a study introduced a Long Short-Term Memory-based Model Predictive Control (LSTM-MPC) method that predicts system behaviours and automatically adapts to varying operational modes without requiring switching strategies^17^. Additionally, advancements in data transmission architectures, such as dual prediction schemes and cloud-edge collaboration, have been proposed to optimize communication bandwidth while maintaining data accuracy^18^. These developments highlight the importance of adaptable command transmission methods in enhancing the efficiency and reliability of industrial process control.

Leveraging Wi-Fi and Virtual Private Networks (VPNs) is pivotal for enhancing the flexibility of industrial operations across diverse geographic locations. Wi-Fi facilitates seamless wireless communication, enabling real-time data exchange and control within industrial environments^19^. It ensures secure and encrypted connections over public networks when integrated with VPNs, safeguarding sensitive operational data from potential cyber threats^20^. This combination allows industries to maintain robust and secure communication channels, irrespective of physical location, thereby optimizing operational efficiency and supporting remote management and monitoring.

Remote control is pivotal in smart factory operations, enabling real-time monitoring and management of manufacturing processes. This capability enhances flexibility, allowing for swift adjustments to production parameters and rapid response to system anomalies^21^. By facilitating remote oversight, manufacturers can optimize resource allocation, reduce downtime, and improve overall efficiency^8^. The integration of remote control systems also supports scalability, as it allows for the seamless expansion of operations without extensive on-site infrastructure^22^.

### Paper organization

The rest of this paper is organized as follows. Section “Methodology” introduces the system under study, including hardware specifics and the setup used for command transmission. Section “New control methods” presents our proposed dynamic command-transmission approaches in detail, outlining single- versus bundled-command methods, their dual-core implementations, and VPN-based solutions. Section “Results” discusses the performance metrics for each method, comparing them to the baseline on-site solution. Section “Discussion” interprets the findings in the context of practical industrial applications, emphasizing the trade-offs among speed, security, and adaptability. Section “Conclusion” summarizes the key contributions, highlighting the importance of secure remote control for modern factory settings and suggesting future research directions. Finally, the Conflict of Interest and Data Availability section clarifies any potential conflicts and provides repository information for data reproducibility.

## Methodology

The Fichertechnik “Multi-Processing Station with 24 V Oven” comprises four key modules: the oven, vacuum gripper, turntable, and conveyor belt. The oven module is equipped with internal and external reference switches, a light barrier, and internal illumination. The vacuum gripper features two reference switches corresponding to the oven and turntable positions and utilizes a vacuum valve to transfer workpieces between the oven and the turntable. The turntable is outfitted with three reference switches for the vacuum gripper, saw, and belt positions, and includes a piston to move the workpiece onto the conveyor. The conveyor belt transports the workpiece to the end of the line, where a light barrier detects its arrival. Additionally, the station is equipped with a 24 V air compressor and four motors.

In this study, the ESP32-WROOM-32 module was employed as the control system. The Multi-Processing Station operates at 24 V DC, whereas the ESP32 operates at 3.3 V DC. To accommodate these voltage differences, a Toshiba BiCD Integrated Circuit (Silicon Monolithic TBD62783APG) was utilized to step down the voltage from 24 to 3.3 V and vice versa, following the same voltage conversion approach used in^23^.

The ESP32-WROOM-32 module, based on the ESP32-D0WDQ6 chip, is a robust WiFi, Bluetooth (BT), and Bluetooth Low Energy (BLE) microcontroller unit (MCU) designed for a wide array of applications, ranging from low-power sensor networks to resource-intensive tasks such as audio streaming and decoding^24^. It integrates dual CPU cores, adjustable clock speeds (80 MHz to 240 MHz), and a low-power co-processor to monitor peripherals while conserving energy. With support for 802.11 b/g/n Wi-Fi and Bluetooth version 4.2, it facilitates both long-range internet access and localized device communication.

In this study, eleven different control methods were employed to operate the station. The time required to complete the process was measured ten times for each control method. The timer starts when the vacuum gripper lowers to pick up the workpiece from the oven position. It then moves to the turntable, places the workpiece down, and the turntable moves to the saw position, where it pauses for one second. Next, the turntable moves to the belt position, where the piston pushes the workpiece onto the belt. The conveyor belt moves the workpiece until it stops upon detection by the light barrier. This detection signaled the end of the time measurement.

### New control methods

Methods 1 to 10 involve the remote control of the ESP32 microcontroller, facilitating interaction with a Python script optimized for efficient command transmission and execution. In these methods, the ESP32 connects to a Wi-Fi network using predefined credentials and establishes an HTTP server on port 80 to listen for incoming requests. This configuration allows the Python script to communicate effectively with the ESP32 by generating URLs that include the microcontroller’s IP address and the designated port for sending commands.

#### Method 0: Direct on-site control

**Fig. 1:**
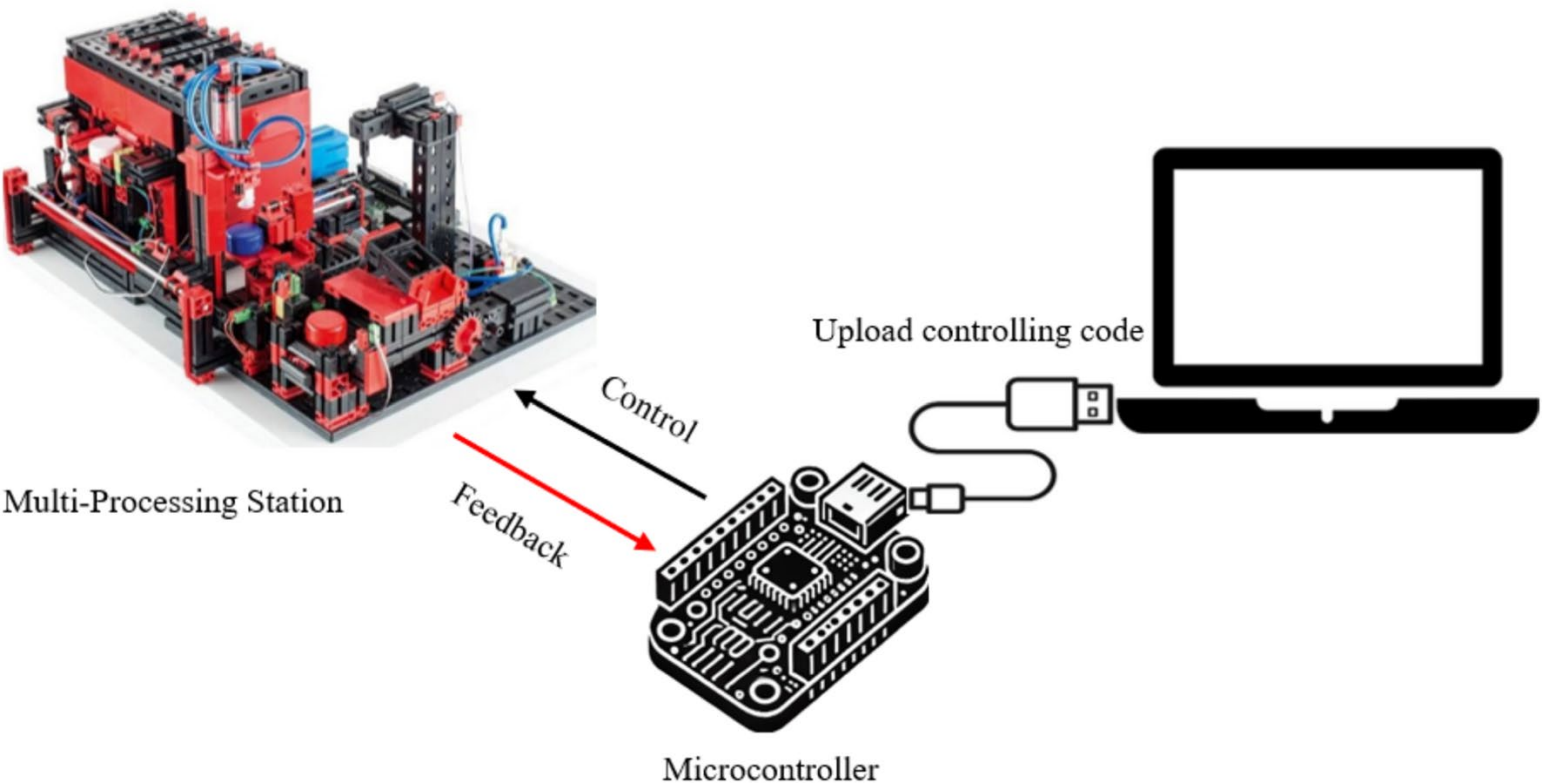
Direct on-site control.

Method 0 serves as a baseline for comparing the time efficiency of the subsequent remote control methods. In this traditional approach, the ESP32 microcontroller is physically present on-site, and the control code is directly uploaded without network communication (Fig. 1). This method provides a reference point for assessing the performance and responsiveness of remote interaction techniques.

#### Method 1: Single command transmission over local network

**Fig. 2:**
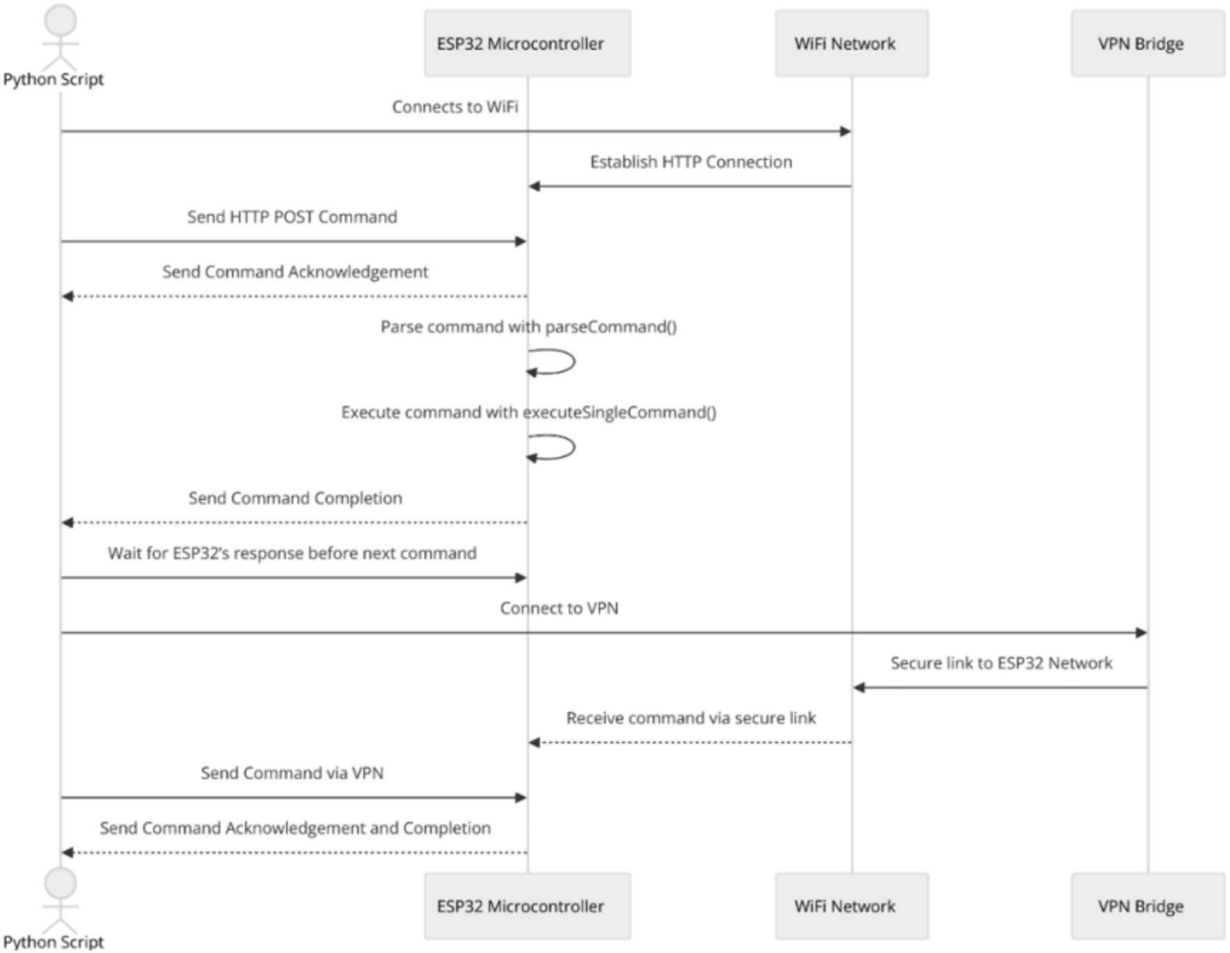
Single command transmission over local network/VPN sequence diagram.

In Method 1, the Python script sends individual commands to the ESP32 via HTTP POST requests over the same local network. The ESP32 continuously checks for client connections within its loop() function. Upon detecting an incoming request, a WiFiClient object is created to manage the connection and reads the HTTP request line by line until a blank line indicates the end of the request. The ESP32 then extracts the command string and invokes a parseCommand function to process control structures such as if and while, breaking the command into executable parts as necessary. Execution is handled by the executeSingleCommand function, which performs actions like changing pin states using digitalWrite and managing delays. After execution, the ESP32 sends an HTTP response acknowledging completion. Upon receiving this response, the Python script knows that the previous command has been successfully processed and can send the following command (Fig. 2).

#### Method 2: Single command transmission over VPN

Method 2 mirrors Method 1 in functionality, with the primary difference being that the Python script and the ESP32 are on different networks. A Virtual Private Network (VPN) is employed to bridge the Python script to the ESP32’s network, enabling communication as if both devices were on the same local network (Fig. 2). This setup allows for remote control without modifying the underlying communication protocol.

**Fig. 3:**
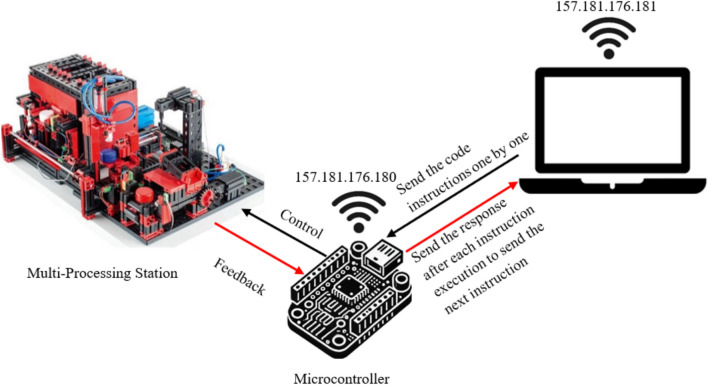
Single command transmission over local network/VPN.

Figure 3 provides the setup used in the Single Command Transmission Over Local Network/VPN (Method 1 and 2)**.**

#### Method 3: Bundled command transmission over local network

**Fig. 4:**
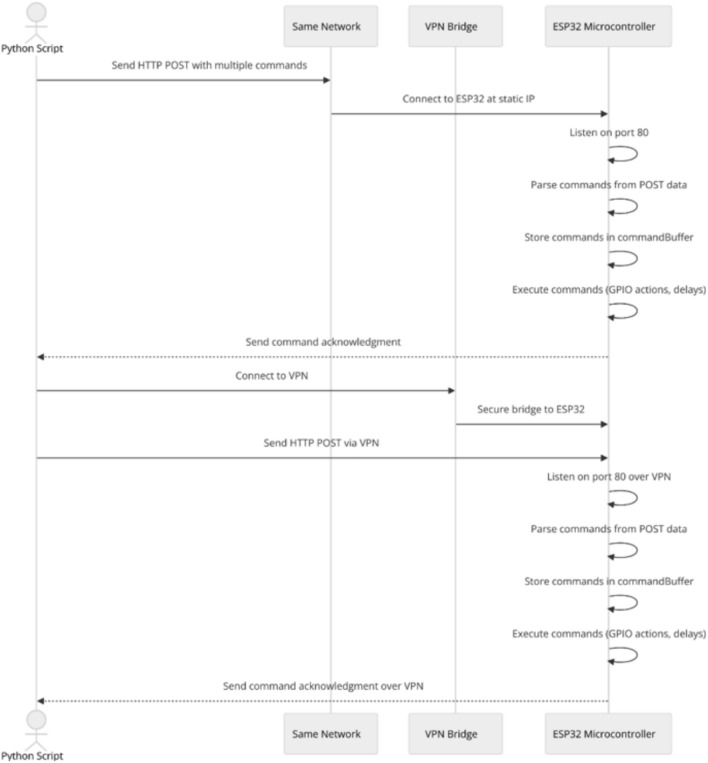
Bundled command transmission over local network/VPN sequence diagram.

In Method 3, the Python script sends multiple commands bundled into a single HTTP POST request over the same local network. The script connects directly to the ESP32 at a static IP address on port 80. Upon detecting a connection, the ESP32 reads the HTTP request headers and the POST data containing all commands combined into a single string, separated by newline characters. This data is passed to a parseCommands function, which splits the string line by line and stores each command in a commandBuffer for sequential processing. The ESP32 executes each command from the buffer in order. The executeCommand function handles commands, supporting control structures such as if statements and while loops, and basic GPIO functions like digitalWrite, digitalRead, and delay. Conditional commands are evaluated in real-time, allowing the ESP32 to respond dynamically to specific pin states (Fig. 4).

#### Method 4: Bundled command transmission over VPN

Method 4 mirrors Method 3, with the addition of a VPN enabling communication between the Python script and the ESP32 across different networks. This configuration allows the Python script to securely access the ESP32 remotely, bridging them as though they are on the same local network (Fig. 4). The VPN ensures secure and stable communication, while the ESP32 handles the bundled command sequence identically to the local setup.

**Fig. 5:**
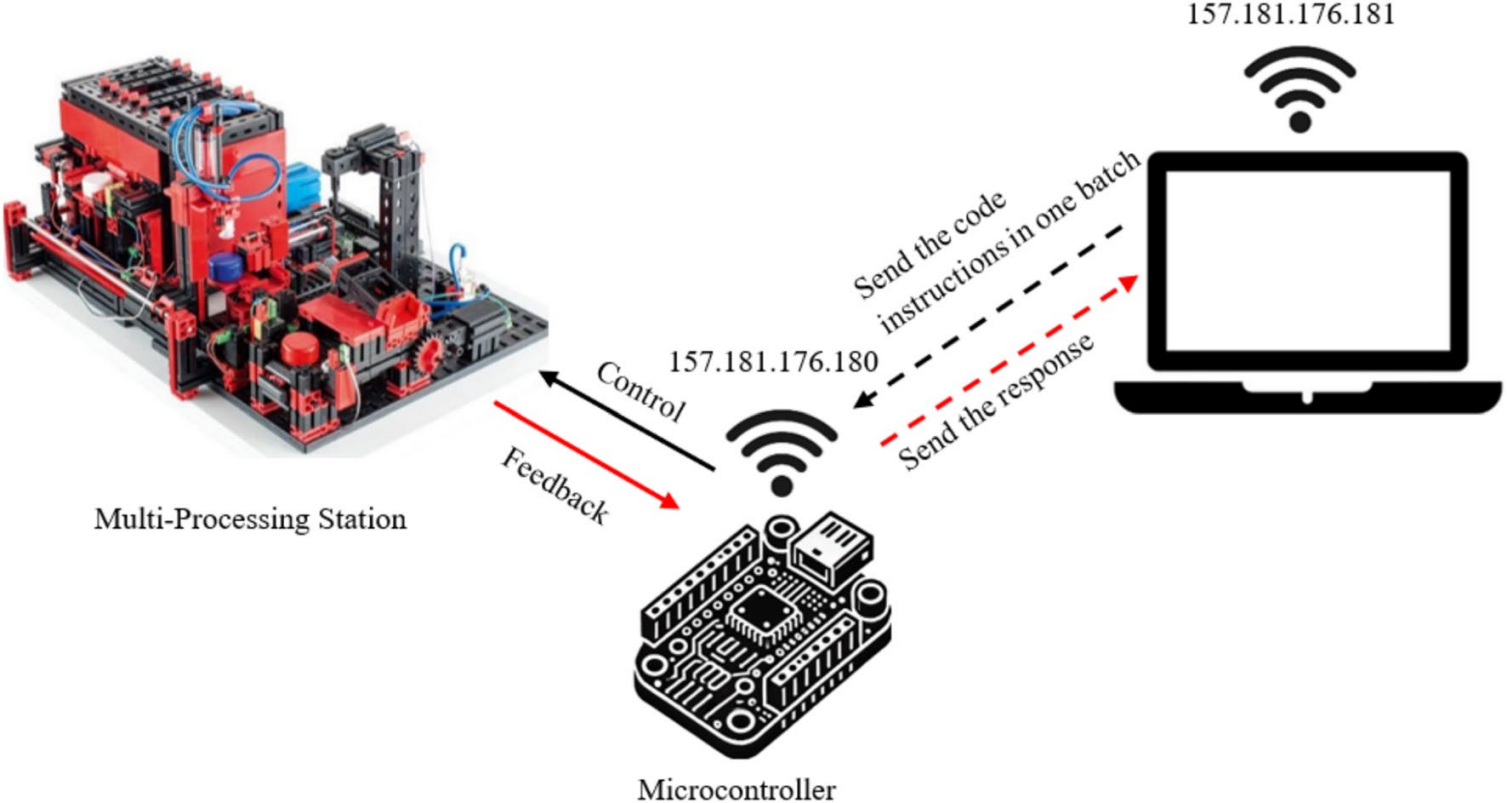
Bundled command transmission over local network/VPN.

Figure 5 provides the setup used in the Bundled Command Transmission Over Local Network/VPN (Method 3 and 4).

#### Method 5: Individual command transmission with dual-core processing over local network

**Fig. 6:**
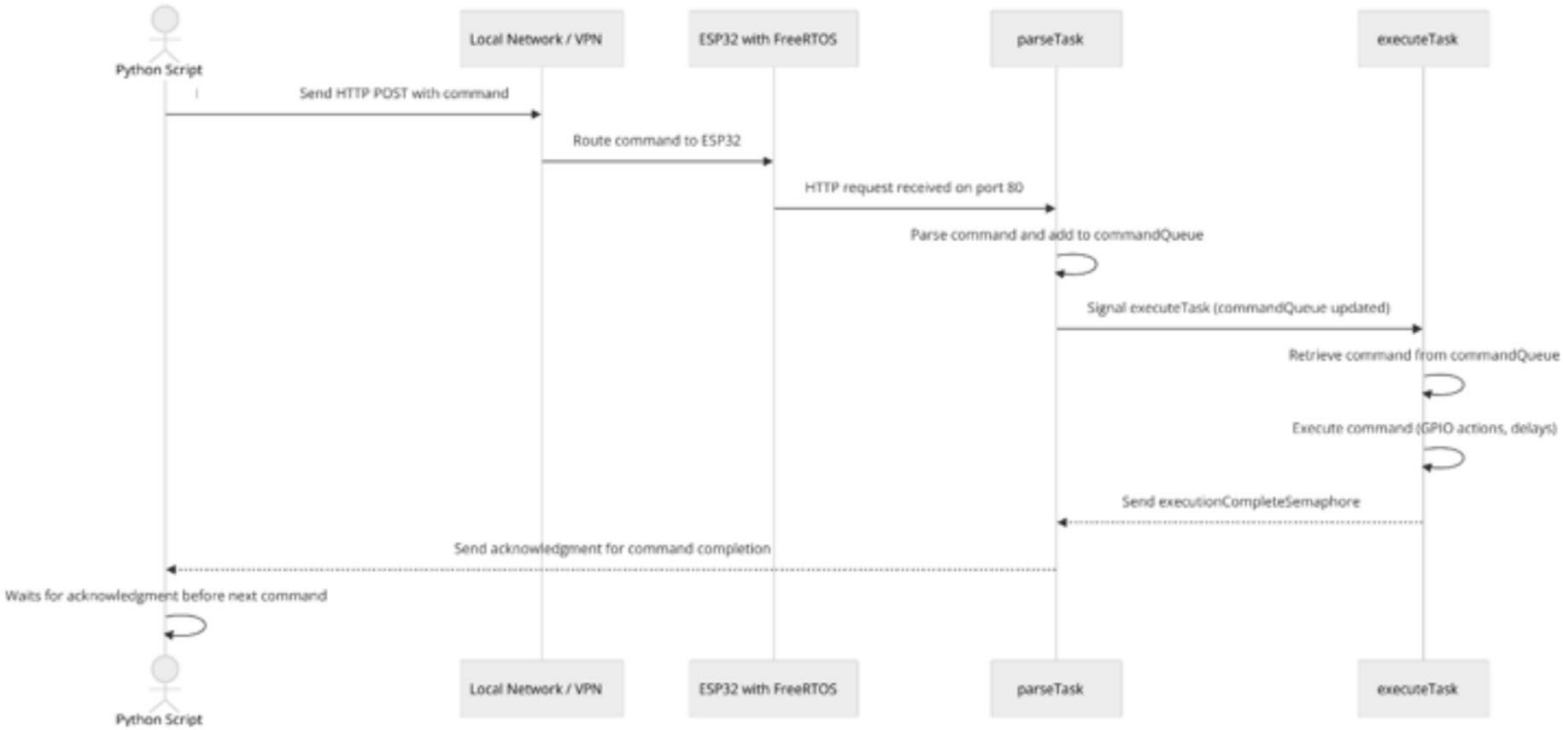
Individual command transmission with dual-core processing over local network/VPN sequence diagram.

Method 5 involves the Python script sending commands individually over the same local network, while the ESP32 processes each command in parallel using FreeRTOS. The ESP32, connected with a static IP, sets up an HTTP server on port 80 to receive commands. Parallel processing is achieved through two FreeRTOS tasks: one for parsing incoming commands (parseTask) and another for executing them (executeTask). This separation allows the ESP32 to handle new commands while executing previous ones. A command queue (commandQueue) and a semaphore (executionCompleteSemaphore) coordinate command flow. The parseTask listens for HTTP requests, extracts commands, and places them into the queue. It waits for a semaphore signal from the executeTask upon command completion to confirm execution to the Python client. The executeTask retrieves commands from the queue, executing them using functions like digitalWrite, digitalRead, and delay, and can handle conditional statements such as if and while (Fig. 6).

On the client side, the Python script sends each command as an HTTP POST request through a send_command(command) function, confirming successful execution before sending the next command. The ESP32’s parallel tasks enable efficient command handling by managing commands individually, with responses sent after each execution (Fig. 6).

#### Method 6: Individual command transmission with dual-core processing over VPN

Method 6 mirrors Method 5, adding a VPN bridging the Python script and the ESP32 across different networks (Fig. 6). This secure VPN connection allows remote command handling, leveraging the same FreeRTOS parallel processing to ensure smooth communication and reliable command execution as in Method 5.

**Fig. 7:**
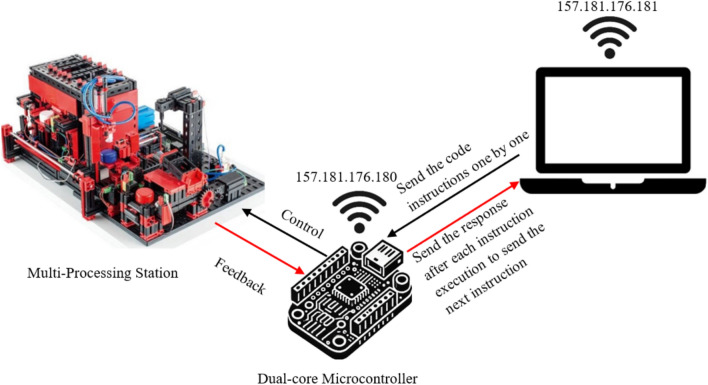
Individual command transmission with dual-core processing over local network/VPN.

Figure 7 provides the setup used in the Individual Command Transmission with Dual-Core Processing Over Local Network/VPN (Method 6 and 7).

#### Method 7: Bundled command transmission with dual-core processing over local network

**Fig. 8:**
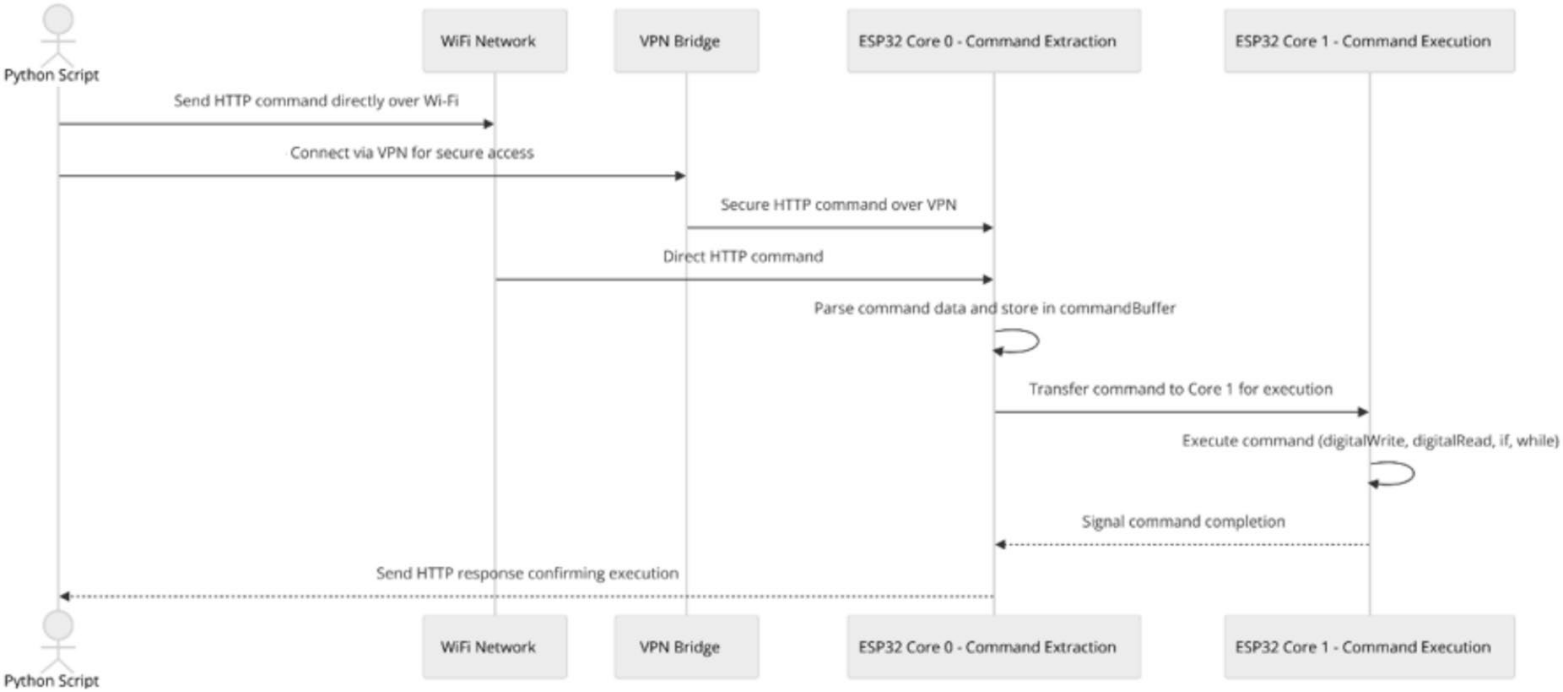
Bundled command transmission with dual-core processing over local network/VPN sequence diagram.

Method 7 involves the Python script sending multiple commands bundled in a single HTTP POST request directly over the same local network. The ESP32 leverages its dual-core functionality to handle command reception and execution in parallel, optimizing responsiveness. Connected to a specified Wi-Fi network with a static IP, the ESP32 runs an HTTP server on port 80. Core 0 manages client connections, reads HTTP requests, and extracts commands, splitting them line by line into a commandBuffer. Core 1 processes each command in sequence, executing actions such as digitalWrite, digitalRead, and handling conditional structures like if and while loops. Once all commands are executed, Core 0 sends an HTTP response back to confirm completion (Fig. 8).

#### Method 8: Bundled command transmission with dual-core processing over VPN

Method 8 mirrors Method 7, with the addition of a VPN enabling communication between the Python script and the ESP32 across different networks (Fig. 8). The VPN allows the devices to communicate on the same local network, maintaining efficient command processing and offering secure remote access to the ESP32, which is ideal for remote operation scenarios.

**Fig. 9:**
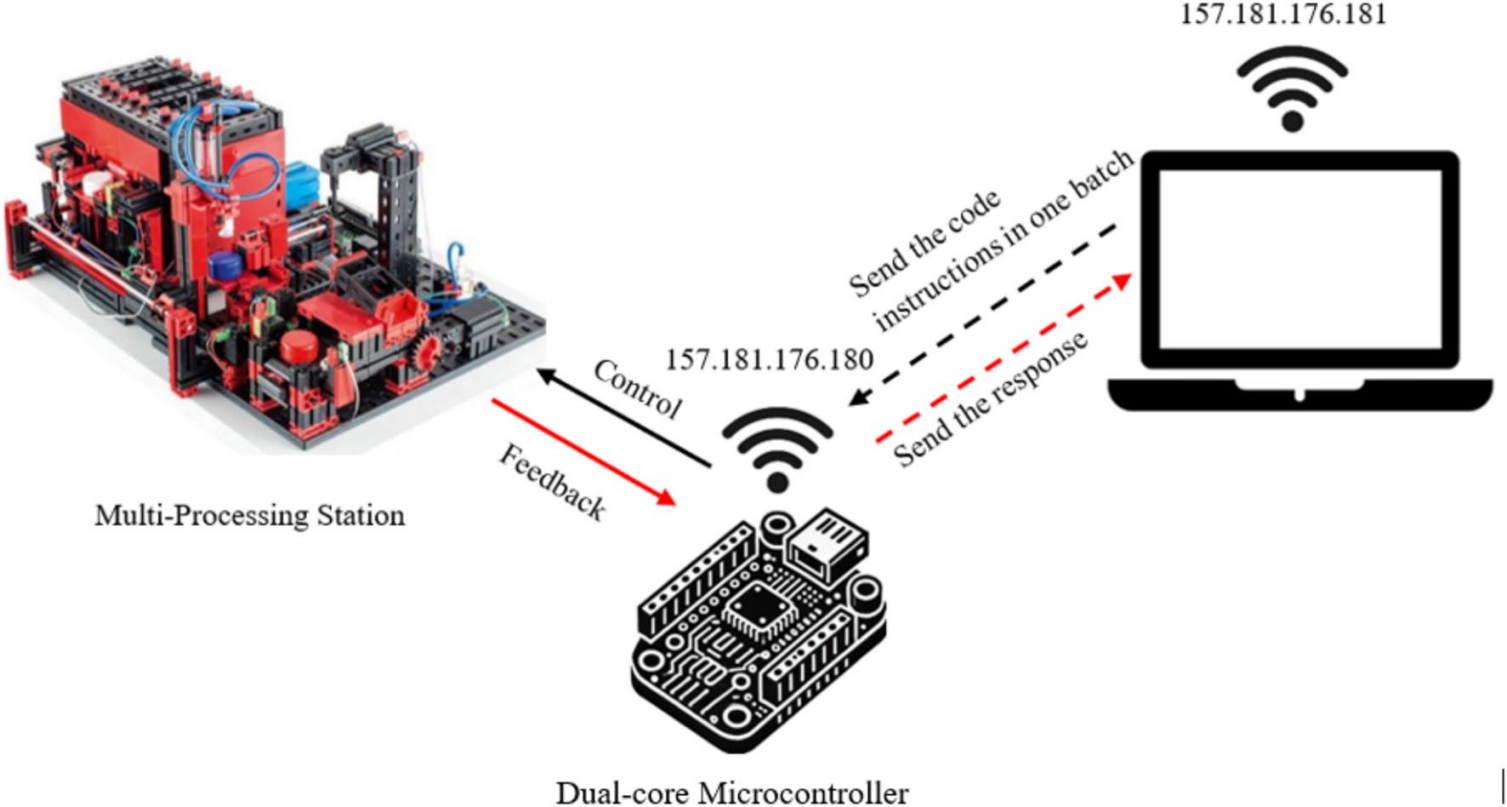
Bundled command transmission with dual-core processing over local network/VPN.

Figure 9 provides the setup used in the Bundled Command Transmission with Dual-Core Processing Over Local Network/VPN (Method 7 and 8).

#### Method 9: Sequential command transmission over local network

**Fig. 10:**
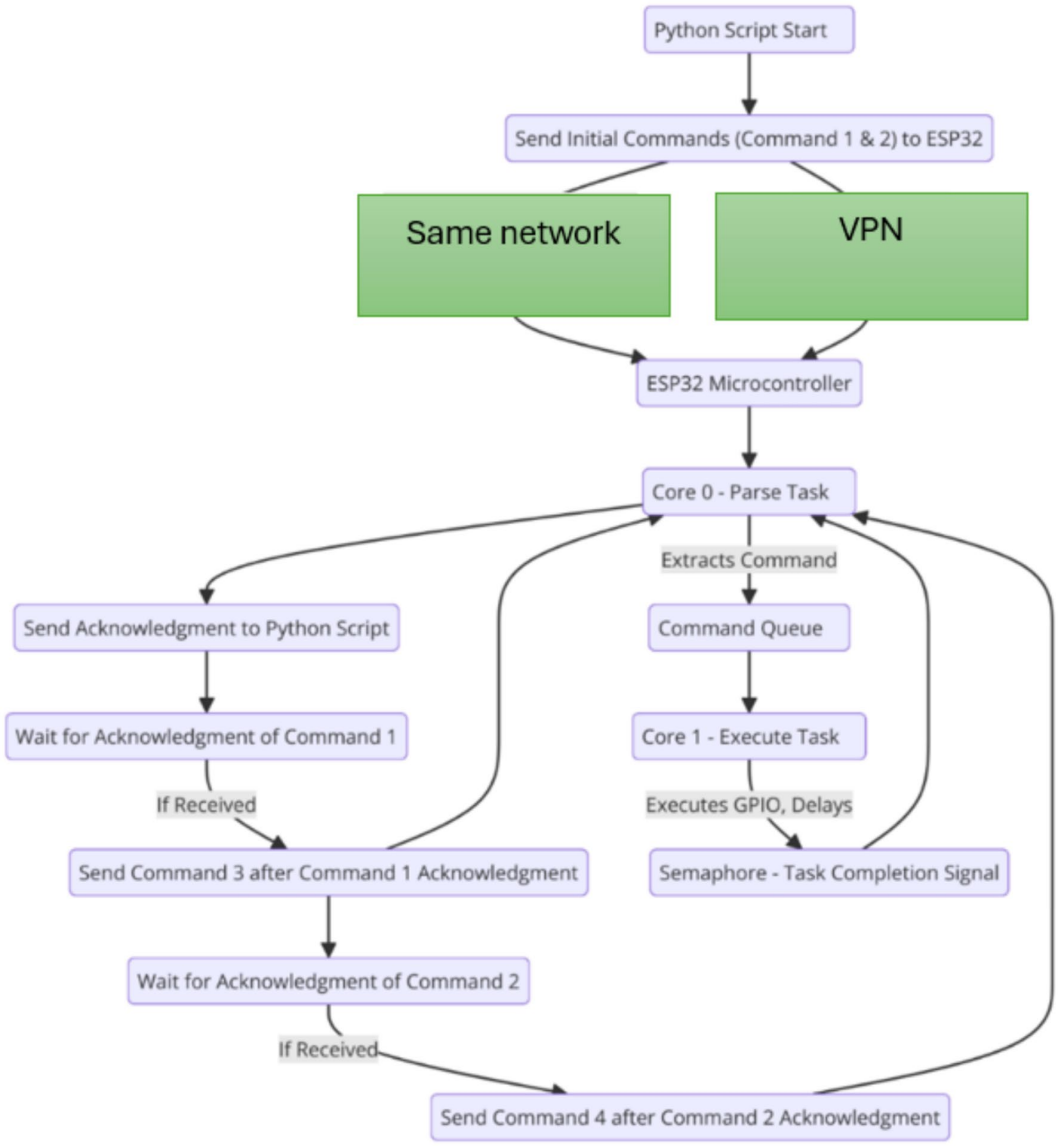
Sequential command transmission over local network/VPN sequence diagram.

Method 9 involves the Python script communicating with the ESP32 over the same local network, sending commands sequentially (creating the possibility of a closed-loop control system ) where the ESP32 uses FreeRTOS for parallel processing. The ESP32, with a static IP, listens for HTTP requests on port 80, managing command handling across two cores to optimize responsiveness and command flow. Upon receiving commands, the ESP32’s Parse Task on Core 0 reads the incoming HTTP request, extracts the first command, and places it in a commandQueue. The Execute Task on Core 1 retrieves and executes this command, signaling completion with a semaphore. After each command executes, the Parse Task sends a response to the Python script, confirming execution and readiness for the next command (Fig. 10).

In the Python script, two commands are sent initially. After the first two commands are sent, the script waits for the ESP32’s response confirming the first command’s completion. Once received, the third command is sent, and upon receiving confirmation for the second command, the fourth command is sent. This back-and-forth sequence continues, ensuring each command is executed in order and acknowledged before proceeding (Fig. 10). This method leverages FreeRTOS to maintain parallel processing on the ESP32, enabling efficient command execution and controlled feedback to the Python script, ensuring smooth operation in a closed-loop system.

#### Method 10: Sequential command transmission over VPN

Method 10 mirrors Method 9, with the addition of a VPN to allow the Python script to communicate with the ESP32 across different networks. The VPN bridges the connection so that the Python script can send commands as though both devices are on the same local network, maintaining the closed-loop control and ensuring secure remote communication.

**Fig. 11:**
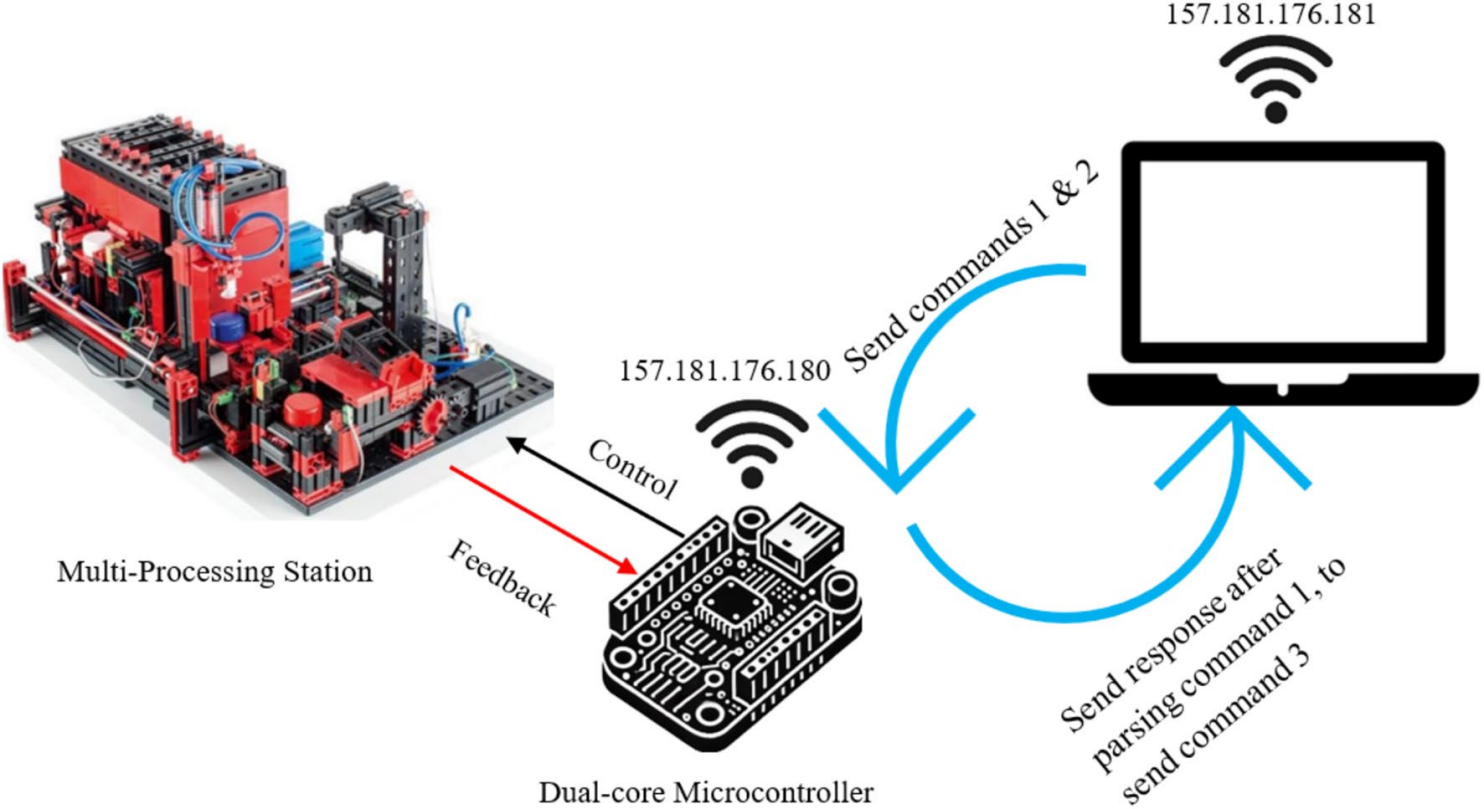
Sequential command transmission over local network/VPN.

Figure 11 provides the setup used in the Sequential Command Transmission over Local Network/VPN (Method 9 and 10).

**Fig. 12:**
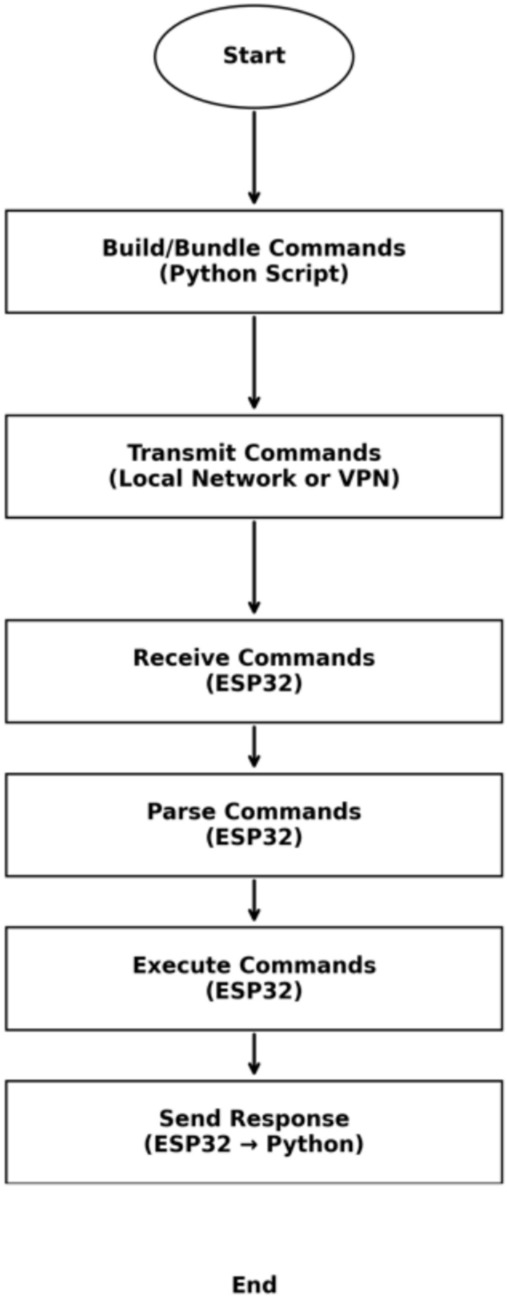
Remote control system flowchart.

To ensure secure and efficient command execution in industrial remote control applications, the proposed system follows a structured communication process between the Python-based control script and the ESP32 microcontroller. This process eliminates the need for preloaded firmware by dynamically transmitting and executing commands in real-time. The Python script constructs and bundles commands, which are then transmitted over a local network or via a Virtual Private Network (VPN) for remote operations. Upon receiving the commands, the ESP32 parses the instructions, executes them accordingly, and sends a response back to the Python script to confirm execution. This closed-loop approach enhances flexibility, security, and adaptability while minimizing the risks associated with storing operational code on the microcontroller. The complete workflow is illustrated in Fig. 12, depicting each stage from command generation to execution and acknowledgment.

## Results

The performance of the ten different methods for controlling an ESP32 microcontroller remotely (Table 1) was evaluated by measuring the time taken to complete a predefined process. Each method was tested ten times to ensure reliability, and the average completion time and the standard deviation were calculated. The results are summarized in Table 2.

**Table 1:** Methods using local network and VPN for ESP32 control.

Communication type	Local network	With VPN connection
Single command	Method 1	Method 2
Grouped commands	Method 3	Method 4
Single command and divided tasks	Method 5	Method 6
Grouped commands and divided tasks	Method 7	Method 8
Sequential commands	Method 9	Method 10

**Table 2:** Average completion time, standard deviation, and relative speed of methods.

Method	Average time (s)	Standard deviation (ms)	Standard deviation (%)	Speed percentage (%)
0	14.6	77	0.53	100
1	22.2	534	0.24	65.7
2	22.6	203	0.9	64.6
3	15.6	82	0.53	93.5
4	15.6	71	0.46	93.5
5	20.6	177	0.86	70.8
6	21	41	0.2	69.5
7	15.7	62	0.39	92.9
8	15.7	162	1.03	92.9
9	17.3	83	0.48	84.3
10	17.5	104	0.59	83.4

In addition to completion times & deviation, and the relative speed of each method was calculated and added to Table 2, which provides a clear comparison of performance efficiency across different methods. The speed is expressed as a percentage relative to Method 0, which serves as the reference method with a speed of 100%. A higher percentage indicates a faster method, while a lower percentage signifies a slower one. For example, a method with 65.7% operates at 65.7% of the speed of Method 0, making it slower, whereas a method with 93.6% is only slightly slower than the reference.

## Discussion

The aim of this research is to develop an appropriate control system for remotely managing industrial processes. Table 2 presents the main characteristics of the different methods compared to Method 0, a traditional direct on-site solution. Method 0 achieved the fastest average completion time (14.6 s) because it operates without any network communication overhead.

**Fig. 13:**
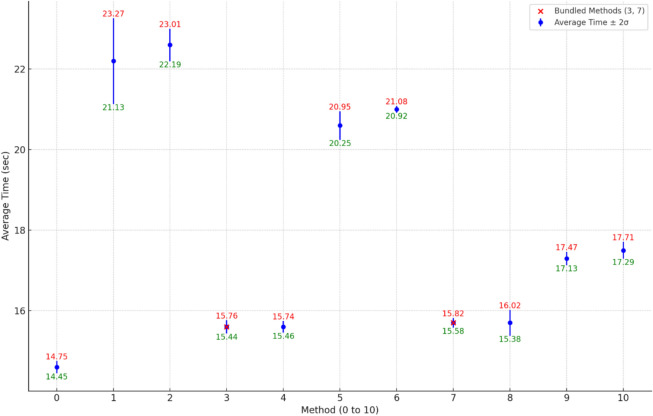
Impact of command bundling on process time: variability analysis with ± 2σ error bounds.

Figure 13 illustrates the impact of command bundling on process time performance, with ± 2σ error bounds labeled as green (lower) and red (upper) for each method. Bundled methods (Methods 3, 4, 7, and 8) demonstrate lower average completion times and tighter variability, closely aligning with the baseline set by Method 0. The small deviations observed in Methods 3, 4, and 7 highlight the consistency of these methods in minimizing delays. Methods 1 and 2, with wider bounds, exhibit higher variability. This strength of bundling commands lies in its ability to improve efficiency and reliability, making it ideal for precision-critical applications.

Dual-core processing in Methods 7 and 8 effectively handles bundled commands with minimal overhead. This indicates that leveraging the ESP32’s hardware capabilities alongside efficient command transmission methods can significantly optimize performance.

Selecting the appropriate method depends on more factors than just communication speed. For instance, Methods 1 and 2 involve transmitting individual commands over local networks or VPNs, making them ideal for systems requiring intermittent control, such as HVAC management or standalone machinery. These methods emphasize simplicity and feedback control, though they incur increased communication delays compared to more advanced approaches. Conversely, bundled command methods (Methods 3 and 4) enhance efficiency by reducing communication overhead, making them well-suited for applications involving rapid, multi-step processes like conveyor systems or irrigation networks where feedback control is infrequently required.

Advanced techniques, such as individual command transmission (Methods 5 and 6) or bundled command processing with dual-core utilization (Methods 7 and 8), excel in dynamic environments with multiple devices, including AGV coordination in warehouses or complex drilling operations. Sequential command methods (Methods 9 and 10) prioritize high precision and reliability, making them optimal for systems requiring closed-loop control, such as logistics, medical devices, or high-accuracy assembly processes where subsequent operation steps highly depend on sensor signals (e.g., actual color, mass, distance).

Among these, Methods 7 and 8 stand out for their optimal balance of performance and adaptability by utilizing dual-core processing. These methods effectively manage bundled commands with minimal overhead, demonstrating that harnessing the ESP32’s hardware capabilities in conjunction with efficient command transmission methods can optimize performance. This approach is particularly suitable for high-speed, complex industrial processes, such as real-time quality control or dynamic remote operations. However, this type of communication is best suited for scenarios with a single possible solution, where feedback is not required at each step. Examples include fast-paced industrial processes like automated assembly lines or continuous production steps that prioritize throughput over interactivity.

Methods 9 and 10 focus on sequential command transmission, ensuring accurate closed-loop control and real-time feedback. These methods utilize the ESP32’s dual-core FreeRTOS to divide tasks between receiving and executing commands, allowing for efficient and error-free processing. Commands are sent one at a time, with each execution confirmed before the next is transmitted, ensuring proper sequencing and reducing the chance of errors. Method 10 builds on this by incorporating a VPN, enabling secure communication between devices across different locations. Although the use of a VPN introduces slight latency, it enhances security while preserving the sequential command flow.

These methods are ideal for applications that require precision, secure remote control, and reliable error management, particularly in industrial automation. This remote solution not only offers flexibility but also protects against intellectual property theft and unauthorized use of production facilities. By keeping the control system and decision-making algorithms off-site, essential commands (such as START and STOP) must be transmitted remotely. As a result, the factory’s machinery cannot operate without the control system, preventing unauthorized production. However, this approach can reduce operational speed by up to 16%, and if the network connection is lost, production comes to a complete halt.

The comparative analysis of ESP32 transmission methods across local network and VPN configurations highlights key considerations for designing a remote control system for factories. Table 3 presents the average time differences between various command transmission methods executed over a local network and a VPN. Each row in the table represents the average difference in execution time across ten tests for a specific method, quantifying the impact of using a VPN on transmission latency. The results demonstrate varying levels of latency introduced by the VPN, with methods involving dual-core processing and sequential transmission exhibiting more significant differences in certain cases. Moreover, the physical distance and the number of active devices were not taken into account in these experiments. These factors can influence communication overhead, which will be addressed in future work.

**Table 3:** Average time differences between command transmission methods over local network and VPN.

Method pairs	Average time differences (s)
Single command transmission	≈ 0.4
Bundled command transmission	≈ 0.0
Individual command transmission with dual-core processing	≈ 0.4
Bundled command transmission with dual-core processing	≈ 0.0
Sequential command transmission	≈ 0.2

### Computational overhead and complexity

While the advantages of flexible remote control are clear, the network-based approach inherently introduces additional computational overhead and system complexity compared to traditional on-site solutions (Method 0). Some of the key factors contributing to overhead include:

1. **Network Latency and Encryption:**
  In Methods 2, 4, 6, 8, and 10, using a Virtual Private Network (VPN) adds an encryption/decryption step, which can increase round-trip times. Although the added latency measured in this study remained within 0.2—0.4 s for most runs, the exact delay can vary under heavier network loads or larger physical distances.
2. **Dual-Core Task Scheduling:**
  Methods 5, 6, 7, 8, 9, and 10 employ FreeRTOS tasks running on separate cores to parse and execute commands in parallel. While this approach significantly boosts responsiveness, it also requires careful scheduling and synchronization. Improperly configured tasks or lock contention can lead to bottlenecks.
3. **Dynamic Command Parsing:**
  Our technique dynamically parses commands upon receipt, allowing for real-time updates and no permanent code storage on the microcontroller. However, this parsing step consumes additional CPU cycles compared to a static, preloaded firmware. The size and complexity of command bundles can further influence the time spent on parsing, particularly in advanced industrial workflows involving conditional structures (e.g., if, while loops).
4. **Implementation Complexity:**
  Distributing logic between off-site control scripts and on-device parsers demands robust error handling, thorough testing, and well-structured software design. Missing or malformed commands can stall the process if not caught by proper exception handling routines. Although this distributed design improves security and adaptability, it may be considered more complex to maintain than a single, on-site control application.

Despite these overheads, many industrial scenarios can tolerate the additional latencies because they gain flexibility, IP protection, and improved adaptability. Furthermore, future optimizations such as hardware-accelerated encryption, streamlined message-passing formats, or partial pre-compilation of certain command libraries can help offset some of the parsing and encryption overhead.

Table 4 provides a comparative overview of recent security methodologies in IIoT and SCADA systems. These methods demonstrate various strengths in enhancing security against cyber threats; however, they often involve high computational complexity, require large datasets, or suffer from scalability issues. In contrast, our approach introduces a lightweight, dynamic command execution technique that minimizes stored code on devices, thereby reducing the attack surface and enhancing security in remote industrial control systems.

**Table 4:** Comparison of security methods in IIoT and SCADA systems.

Proposed method	Pros	Cons
Sliding principal component and dynamic reward reinforcement learning for IIoT attack detection^25^	Adaptive learning-based detection; enhances security against evolving threats	High computational overhead; requires continuous model updates
AI-driven lightweight blockchain security model for IIoT^26^	Efficient security and privacy preservation; reduces latency in authentication	Blockchain integration increases system complexity; potential scalability issues
ZESO-DRKFC model for smart grid SCADA Security^27^	High efficiency in anomaly detection; energy-efficient	Sensitive to parameter tuning; may require high-quality training data
Learning algorithm-based security enhancement in SCADA networks^28^	Improves security in industrial appliance integration; adaptable learning mechanisms	Performance degradation under high network traffic
Multifacet clustering and optimization-based SCADA classification^29^	Enhances SCADA resilience against cyber threats	Computationally intensive; requires real-time updates
RPCO-BCNN mechanism for attack detection and classification in SCADA systems^30^	High accuracy in detecting malicious activities; adaptable to dynamic environments	Training complexity; requires large datasets

Limitations of Prior Approaches and Comparative Algorithm Discussion.

Although prior studies^6–8^ have effectively demonstrated remote industrial monitoring using microcontrollers, many of these solutions depend on preloading the operational code onsite. This approach poses two key challenges: (1) Security risks, as static code can be reverse-engineered or misused by unauthorized parties, and (2) Limited adaptability since frequent firmware updates are necessary when production demands change. Some of these conventional models utilize only single-step or single-loop control methods, which can be susceptible to network latency and may not offer real-time adaptability in complex manufacturing processes^11,17^.

Additionally, alternative security-focused frameworks (see Table 4) rely on methods such as blockchain-based authentication^26^ or machine-learning-driven anomaly detection, which enhances data confidentiality but often increases computational overhead potentially hindering real-time performance in large-scale deployments. Likewise, advanced algorithmic strategies such as Model Predictive Control (MPC) and neural network-based controllers^11,17^ excel at dynamic decision-making but can require substantial processing resources, making them less practical on resource-constrained devices or under volatile network conditions.

Our proposed dynamic command transmission method is designed to mitigate these limitations by removing the need to store proprietary code locally. This not only reduces vulnerabilities to reverse engineering but also allows for immediate adaptation to evolving production requirements without reinstalling firmware. By bundling or sequentially transmitting commands based on the operational context, we demonstrate that real-time performance is achievable even under dual-core and VPN conditions. Hence, in comparison to existing approaches, our system provides a better balance between security, adaptability, and computational efficiency, rendering it well-suited for modern, globally distributed smart factory environments.

### Limitations and future work

While the proposed dynamic command transmission method enhances security, flexibility, and adaptability in industrial remote control, it has several limitations that must be considered when deploying in real-world settings. Addressing these challenges is essential for optimizing system reliability and guiding future research directions.

1. **Dependence on Network Stability**
  The system relies on stable network connectivity to transmit executable commands from the remote control server to the ESP32. In environments with unstable networks, such as remote industrial sites with weak infrastructure, the system may experience delays or failures in command execution. A loss of network connectivity results in complete production halts since no operational code is stored locally. Future work could explore hybrid models that retain limited fallback routines locally while maintaining the security benefits of dynamic execution.
2. **Real-Time Constraints and Latency**
  Despite dual-core processing and command bundling techniques that optimize execution, the remote execution of commands introduces inherent network latency. Industrial applications requiring strict real-time constraints, such as high-speed robotic control or time-sensitive production lines, may be affected by variations in command execution timing. Advanced buffering techniques or low-latency network protocols could be explored to mitigate this effect.
3. **Security–Performance Trade-Off**
  The use of VPN encryption ensures secure command transmission but introduces additional computational overhead. Under heavy network load or long-distance communication scenarios, the encryption-decryption process may add small but non-negligible delays. Future research may evaluate alternative lightweight encryption methods that maintain security without significantly impacting real-time performance.
4. **Loss of Local Autonomy**
  Since commands are executed dynamically and erased post-execution, the system lacks local decision-making autonomy. In cases of network failure or cyberattacks, operations cease entirely. A potential solution is to implement a minimal emergency execution framework that retains essential safety and maintenance commands without compromising security.
5. **Increased Software Complexity**
  Unlike traditional on-site systems, which rely on static preloaded firmware, this approach requires real-time command parsing and error handling, increasing software complexity. This may necessitate advanced debugging tools and improved software design practices. Future research could focus on optimizing parsing algorithms to reduce overhead while maintaining security and adaptability.

These limitations highlight areas for potential refinement and improvement. Future research could explore alternative command transmission architectures, more efficient encryption models, and hybrid execution frameworks to further enhance system resilience and performance.

## Conclusion

This research demonstrates the effectiveness of implementing a novel dynamic command transmission approach for remote industrial control using the ESP32-WROOM-32 microcontroller. Unlike traditional on-site methods, this approach eliminates stored operational code by dynamically transferring, executing, and deleting commands, thereby enhancing both precision and security. The study evaluated ten distinct control methods, comparing single, bundled, and sequential command transmissions over local networks and VPNs, both with and without dual-core processing.

The findings indicate that bundling commands and utilizing dual-core processing (Methods 7 and 8) significantly optimize performance by reducing latency and improving reliability, making these methods ideal for high-speed, complex industrial operations. Methods that leverage sequential command transmissions (Methods 9 and 10) proved suitable for precision-critical applications, offering robust closed-loop control. Despite the added latency from VPN usage, its secure communication capabilities underscore its utility for geographically distributed industrial systems.

Ultimately, the study emphasizes that selecting the appropriate command transmission method depends on specific application requirements, balancing simplicity, precision, efficiency, and security. This research establishes a versatile framework for advancing smart factory operations, particularly in environments that demand secure, flexible, and efficient remote control solutions.

**Fig. 14:**
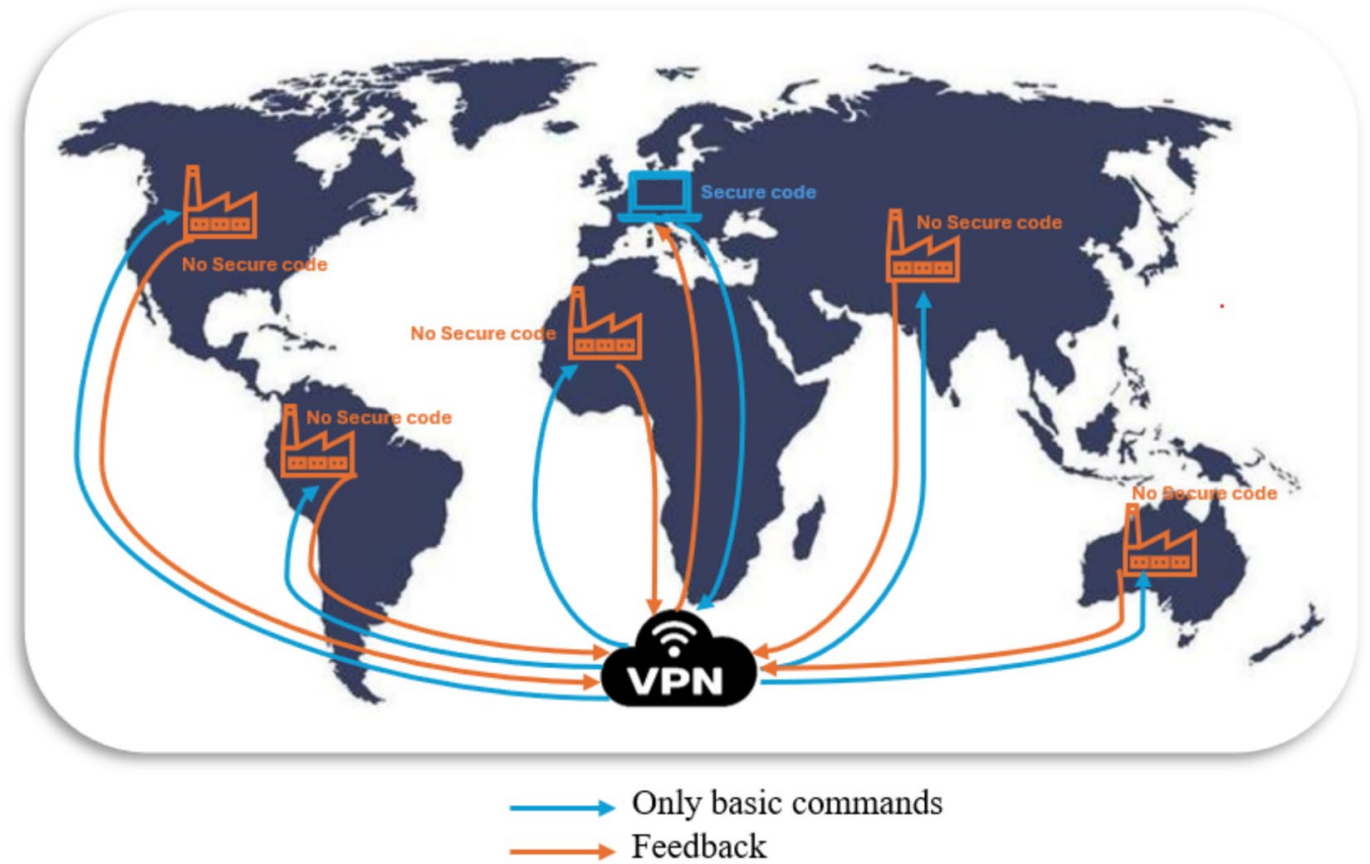
Global summary of secure command transmission in industrial networks using VPN.

Figure 14 illustrates the primary objective of this study: enabling real-time operational code transmission from the central control hub to factories while ensuring feedback from each factory. The figure emphasizes that no operational code is stored locally at the factories, enhancing both security and control efficiency.

## Data Availability

The data and codes that support the findings of this study are available in the below Repository. https://github.com/anasabualhaija/Research-2.git.
